# Dynamic molecular portraits of ion-conducting pores characterize functional states of TRPV channels

**DOI:** 10.1038/s42004-024-01198-z

**Published:** 2024-06-01

**Authors:** Yury A. Trofimov, Nikolay A. Krylov, Alexander S. Minakov, Kirill D. Nadezhdin, Arthur Neuberger, Alexander I. Sobolevsky, Roman G. Efremov

**Affiliations:** 1 Shemyakin-Ovchinnikov Institute of Bioorganic Chemistry, Russian Academy of Sciences, Moscow, Russia; 2 M.V. Lomonosov Moscow State University, Moscow, Russia; 3 Department of Biochemistry and Molecular Biophysics, Columbia University, New York, NY USA

**Keywords:** Molecular modelling, Transient receptor potential channels

## Abstract

Structural biology is solving an ever-increasing number of snapshots of ion channel conformational ensembles. Deciphering ion channel mechanisms, however, requires understanding the ensemble dynamics beyond the static structures. Here, we present a molecular modeling-based approach characterizing the ion channel structural intermediates, or their “dynamic molecular portraits”, by assessing water and ion conductivity along with the detailed evaluation of pore hydrophobicity and residue packing. We illustrate the power of this approach by analyzing structures of few vanilloid-subfamily transient receptor potential (TRPV) channels. Based on the pore architecture, there are three major states that are common for TRPVs, which we call α-closed, π-closed, and π-open. We show that the pore hydrophobicity and residue packing for the open state is most favorable for the pore conductance. On the contrary, the α-closed state is the most hydrophobic and always non-conducting. Our approach can also be used for structural and functional classification of ion channels.

## Abstract (web summary)

Ion channels are known to be important actors in cellular signaling, and their functions correlate to their conformational states, however, detailed function–conformation relationships remain underexplored. Here, the authors use a molecular modeling-based approach to assess water and ion conductivity along with pore hydrophobicity and residue packing to characterize the ion channel structural intermediates, revealing three major states of vanilloid-subfamily transient receptor potential channels.

## Introduction

Some of the most important actors of cellular signaling – ion channels – function by switching between different conformational states^1^. Understanding the relationship between these states is necessary to decipher the molecular mechanism of ion channel function and requires structural information about conformational intermediates, including open and closed states, as well as knowledge about their physiological function. Of particular interest is the molecular organization of the ion channel pore that determines ion channel conductance and gating. When studying this protein region, we assess its ability to interact with water molecules and ions, polar/non-polar properties of the channel walls, as well as packing and conformational dynamics of the pore-lining protein segments, including transmembrane (TM) helices.

**Fig. 1:**
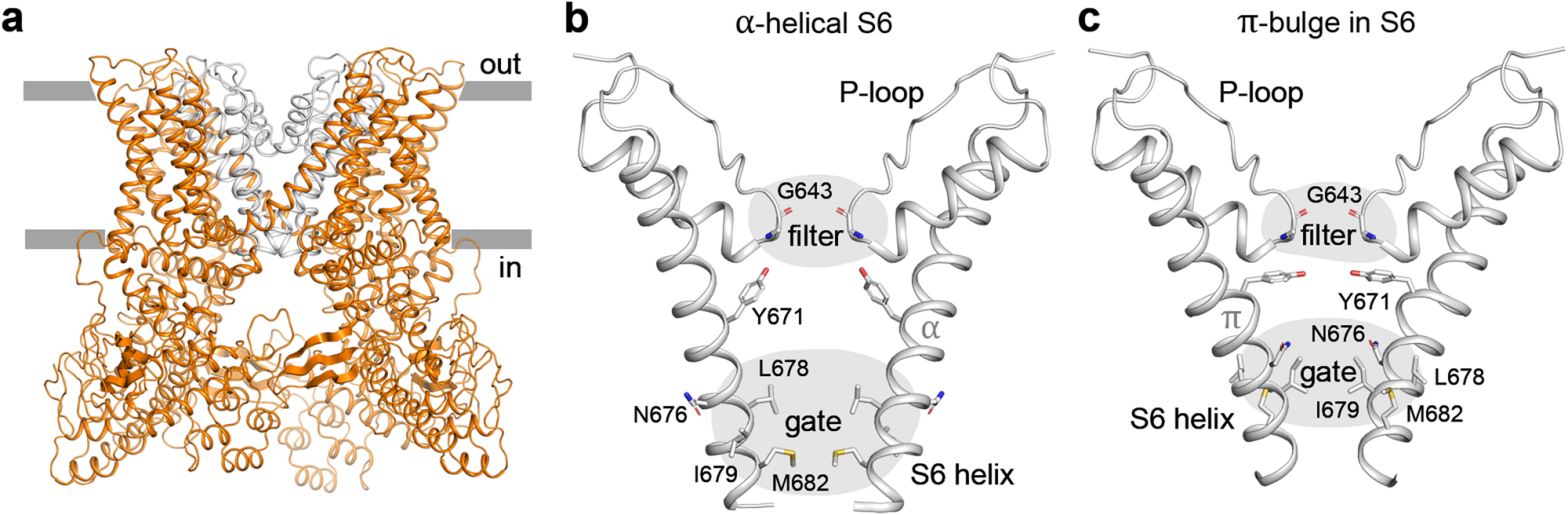
The TRPV structure and its pore on the example of rat TRPV1. **a** – The homotetramer of rat TRPV1 (PDB ID 7L2P) shown in orange, with its ion-conducting pore shown in gray. Gray bars indicate the membrane boundaries. **b**, **c** – Rat TRPV1 pore structure with α-helical (**b**) and π-bulge-containing (**c**) conformation of S6 (PDB ID 7MZD and 7L2P). Only two of four subunits are shown, with the residues lining the pore shown as sticks and labeled. The positions of π-bulge segment, selectivity filter and activation gate are also labeled. Two conformations differ in the structure of activation gate: residues I679 form the narrow constriction when S6 contains the π-bulge, while residues L678 and M682 when S6 is α-helical.

Obtaining structural data for ion channels, especially for their different conformational states, is a challenging task, particularly since the channel protein extraction and purification often leads to the energetic favoritism of one or few discrete states, from which the protein can only be released using carefully determined experimental conditions (appropriate physico-chemical stimuli, TM domain environment, etc.). One of the few exceptions is vanilloid-subfamily transient receptor potential (TRPV) channels, which in the recent 10 years have been characterized by a number of structural models. TRPV channels are permeable to cations and responsible for remarkably diverse biological functions, including nociception, thermosensation, and body temperature regulation^2,3^. The TRPV subfamily consists of six members: four non-selective thermosensitive channels^4^ – TRPV1 (activated at temperatures >42 °C), TRPV2 (>52 °C), TRPV3 (>31–39 °C), and TRPV4 (>22–42 °C) – and two Ca^2+^-selective non-thermosensitive channels – TRPV5 and TRPV6. TRPV1-4 and TRPV5-6 share 40–50% and 75% sequence identity within their groups, while only ~30% between the groups^5^. TRPV channels predominantly function as domain-swapped homotetramers with similar molecular architecture, although a pentameric assembly of TRPV3 channel with a dilated pore has recently been reported^6^. Each TRPV channel subunit consists of a TM-domain, which is composed of six helices (S1-S6) and a re-entrant pore loop (P-loop) between S5 and S6, flanked by cytosolic N- and C-terminal domains. The ion-conducting pore is in the middle of the tetrameric assembly of the TM-domains contributed by individual subunits, with its extracellular region lined by the extended portions of the P-loops and intracellular region lined by the C-terminal portions of S6 helices and hydrophobic residues at the S6 bundle crossing forming the activation (lower) gate (Fig. 1).

Structures of many wild-type or mutant TRPV channels have been solved in different states in the presence or absence of (ant-)agonists. The corresponding conformations have been termed the open, activated, closed, inactivated, desensitized, and inhibited states. The functional state of the pore (conductive or non-conductive) is typically characterized by the minimal pore radius in the gate region (*R*_gate_). If *R*_gate_ is greater than the radius of a hydrated ion, the pore is often considered open. However, the conductivity properties of a nano-dimensional pore are determined not only by its lumen, but also by the chemical properties of its surface. If a hydrophobic pore becomes sufficiently narrow (radius ≤ 4 Å), the corresponding region can undergo spontaneous dehydration, creating an effective energetic barrier for ion permeation and essentially closing the channel for ion transport without substantial pore constriction^7^. Such narrow regions are often called hydrophobic gates, suggesting that many of the available ion channel structures may represent non-conducting states^8^. Additional computational tools can be used to aid functional interpretation of the experimental structures^9^.

All available TRPV structures can be divided into two classes: with entirely α-helical pore-lining S6 and S6 containing a short π-helical segment (π-bulge) located just above the activation gate^10^ (Fig. 1). While both classes presumably include conducting and non-conducting states, their gates are quite different. When S6 is entirely α-helical, leucine and methionine residues form an 8–10-Å-long hydrophobic pore constriction. The α → π transition results in *a* ~ 100° rotation of the lower part of S6^11^. This conformational change alters the pore’s hydrophobic constriction (gate), which is now only 5 Å-long and is contributed by an isoleucine residue. The functional importance of the latter was confirmed experimentally by showing that the double substitution I679A + A680G makes rat TRPV1 constitutively open^12^, while I715N increases the open probability of rat TRPV4^13^. Despite numerous proposed TRPV channel gating mechanisms^5,10,13–17^ and abundance of functional and cryo-EM data, the role of the α → π transition and characteristics of the gate region in channel gating has remained enigmatic.

In this study, we performed quantitative evaluation of physicochemical pore properties for three representative TPRV channels that have been characterized structurally at high resolution in different functional states – rat TRPV1^14^, mouse TRPV3^15,18^ and human TRPV6^19,20^ (Table 1). For all these TRPV channels, we calculated “dynamic molecular portraits” (DMP) of their pores^21^. We revealed that there are three major states of the TRPV pore: α-closed, π-closed, and π-open, that are common for each of these proteins and characterized by specific distribution of hydrophobicity at the gate region and contact interfaces of the pore-forming helices. Other conformational states of TRPV channels appear to be intermediates between the three major states.

**Table 1:** The list of TRPV structures studied in this work

	PDB ID	Description	*R*_gate_, Å	S6-helix conformation	Naming in this work	Refs.
Rat TRPV1 minimal	7L2W	RTX + NMDG bound open state	2.9	π-bulge	π-open-V1	^14^
	7L2P	Closed apo state	0.6	π-bulge	π-closed-V1	^14^
	7MZC	RTX bound closed state	1.0	π-bulge	π-semiopen-V1	^14^
	7MZD	RTX bound closed state	0.5	α-helix	α-closed-V1	^14^
	7L2U	DkTx bound open state	1.2	α-helix	α-open-V1	^14^
Mouse TRPV3	7MIO	Open state at 42 °C	2.8	π-bulge	π-open-V3	^15^
	7MIN	Closed state at 42 °C	0.9	π-bulge	π-closed-V3	^15^
	7MIL	Sensitized state at 42 °C	1.7	π-bulge	π-semiopen-V3	^15^
	6PVL	Closed state at 42 °C	0.7	α-helix	α-closed-V3	^18^
Human TRPV6	7S88	Open apo state	2.9	π-bulge	π-open-V6	^20^
	6E2F	Calmodulin bound closed state	1.3	π-bulge	π-closed-V6	^19^
	7S8C	Econazole bound closed state	0.7	α-helix	α-closed-V6	^20^

*R*_gate_ – the minimal pore radius in a gate region.

## Results

### “Dynamic molecular portrait” approach

Each DMP represents combined 2D/3D distributions of physicochemical properties of the pore-forming TM domains and of pore-filling water and ions. The following parameters were evaluated as DMP components: (i) channel permeability to water and ions, (ii) structural and hydrophobic organization of the pore domain, and (iii) packing quality of the pore-forming helices. We estimated whether water and ions can pass through the ion channel pore (i) during all-atom molecular dynamics (MD) simulations. Structural and hydrophobic pore organization (ii) was evaluated by 2D mapping of hydrophobicity of the entire pore surface or the surface of the pore-forming S6-helix at the gate region using the molecular hydrophobicity potential (MHP) calculations^22–24^. In this case, each atom of the molecular system was characterized by a specific hydrophobicity constant and the sum of individual atomic contributions was plotted on the pore surface. Spatial distributions of non-polar and polar regions in various functional states were then compared across different TRPV channels. Finally, the residue packing parameters for the pore gate region (iii) were evaluated using the 3D–1D profile method^25^. Here, the contribution of each residue in the TRPV channel pore to the quality of microenvironment was quantified using a scoring function based on the analysis of well-packed protein models^26^. The resulting 3D distribution of residues in favorable and unfavorable microenvironments characterized the relative stability of the corresponding channel conformation. Combined outcomes of the three approaches yielded DMPs that characterize the relationships between structural, dynamic, hydrophobic, and packing properties of ion channel pores and their functional states.

### State-dependent changes in hydrophobic organization of the TRPV1 pore

Conformational intermediates between the fully closed and open states of TRPV1 were captured in a series of structures characterized by α-helical or π-bulge-containing S6 and by different extent of pore occlusion^14^. We analyzed three states with π-bulge in the middle of S6 – the fully open (π-open-V1), closed (π-closed-V1), and intermediately open (π-semiopen-V1) gate – and two states with α-helical S6 – the closed (α-closed-V1) and open (α-open-V1) gate (Table 1).

**Fig. 2:**
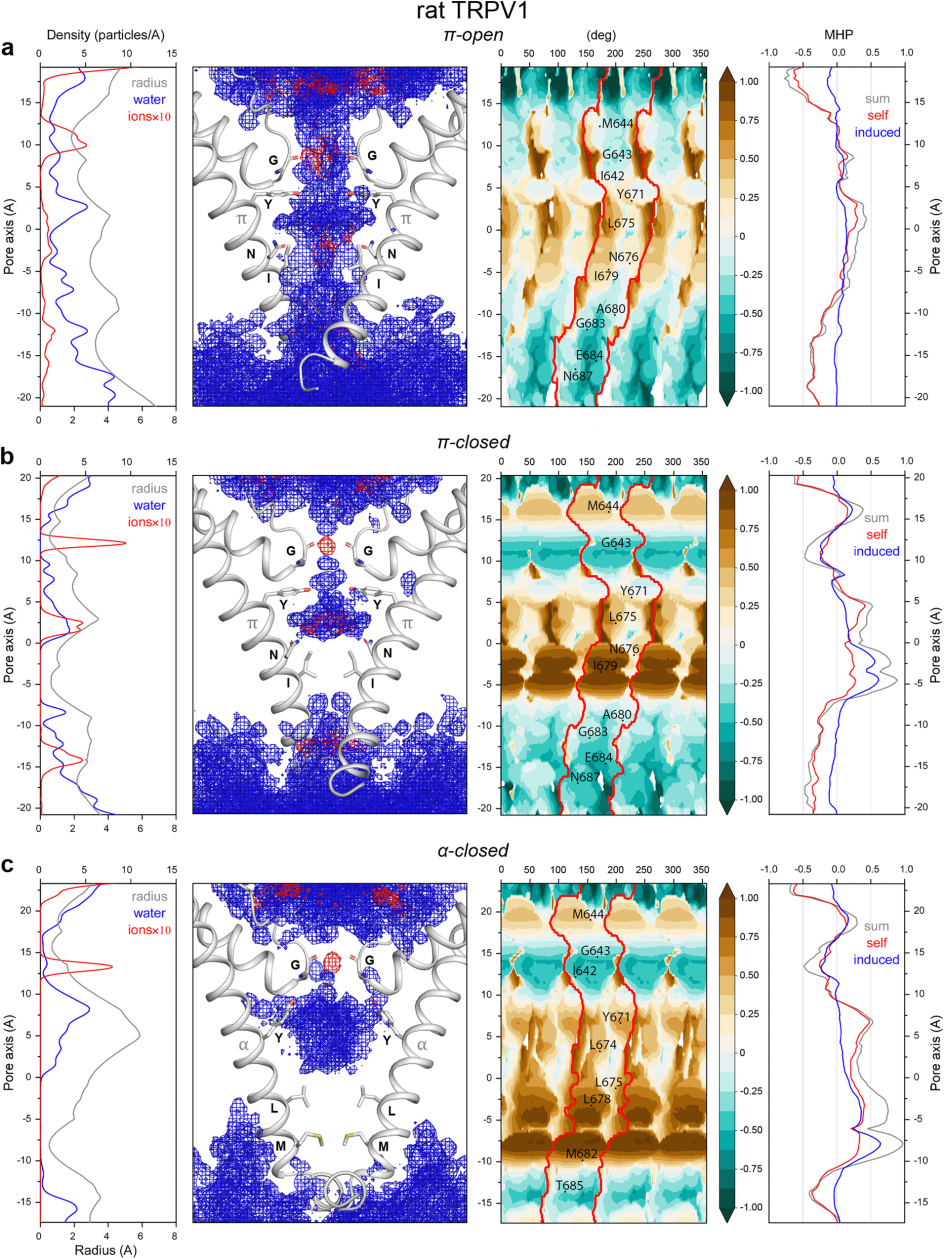
Pore properties of rat TRPV1. **a** – open state with π-bulge conformation of S6 helix (π-open-V1), **b** – closed state with π-bulge conformation of S6 helix (π-closed-V1), **c** – closed state with α-helical conformation of S6 helix (α-closed-V1). The panels in each row from left to right: (1) Density profiles of water molecules and Na^+^ ions averaged over MD simulation (blue and red lines, correspondingly) and profile of the pore radius in the initial structure (black line). (2) Density distributions of water and Na^+^ ions averaged over MD simulation (blue and red meshes, correspondingly) imposed on the initial protein structure (two of four subunits are shown), with residues lining the pore shown as sticks and labeled. (3) Cylindrical projection of sum MHP distribution over the pore surface composed of four protein subunits (MHP maps), blue-green color fills the hydrophilic areas and brown — the hydrophobic ones, coloring scheme for MHP is shown on the right, MHP values are given in octanol-water log P units, the area filled by one of the four subunits is marked by a red curve, the residues lining the pore are marked on a map, cryo-EM protein structures listed in Table 1 are used for mapping. (4) Profiles of self MHP of protein subunits (red line), MHP induced on subunits by neighbor subunits (blue), and the sum of self and induced MHP (black). In each row, all panels are aligned along the pore axis.

MD simulations showed that the pore of π-open-V1 is fully hydrated and conducts ions (Fig. 2a). The pore of π-closed-V1 has two narrow regions. While the selectivity filter formed by G643 can accommodate partly dehydrated ions, the gate formed by I679 stayed non-permeable to water and ions throughout the entire MD simulations (Fig. 2b). We used the initial cryo-EM structures to map hydrophobicity of the pore by projecting MHP on the surface of a cylinder with the axis matching the axis of the channel pore (see “Methods” section). In the resulting maps, blue-green and brown colors highlight hydrophilic and hydrophobic areas, respectively. Integration of the 2D map over the cylinder rotation angle provided 1D MHP profiles of three types: (1) self MHP of protein subunits, (2) MHP induced by neighboring subunits, and (3) the sum of the two. The MHP maps for π-open-V1 and π-closed-V1 are consistent with our previous analysis^24^: TRPV1 pore contains a “hydrophobic belt” lined by Y671, L675, N676, and I679 residues (the sum MHP in this region > 0) and two hydrophilic vestibules above and below the belt (Fig. 2a, b). The selectivity filter is hydrophilic because the carbonyl oxygen atoms of G643 point towards the pore, while the gate in the closed state is a highly hydrophobic 5 Å-long region lined by the non-polar side chains of I679. The high values of MHP in this region are due to the considerable contribution of the induced MHP (50-75% of the sum MHP) caused by tighter packing of gate residues in the closed state compared to the open one. Despite the larger gate lumen in π-semiopen-V1, the behavior of water and ions as well as MHP distribution looked similar to π-closed-V1, with the water-free and highly hydrophobic gate formed by I679 (Supplementary Fig. 1a).

In α-closed-V1, the gate region lined by L678 and M682 is not hydrated during MD simulations (Fig. 2c). The MHP distribution is similar to π-closed-V1 at the selectivity filter, while the length of the highly hydrophobic gate region increases to 10 Å. Although the lumen of the α-open-V1 pore appears to be large enough for dehydrated ion conductance (*R*_gate_ ≈ 1.2 Å) and the corresponding structure (PDB ID 7L2U) was proposed to represent an open state^14^, the gate formed by L678 and M682 was not hydrated or permeable throughout the entire MD simulations. According to our estimates, hydrophobicity of the α-open-V1 pore is reduced only by 20–30% compared to α-closed-V1 (Supplementary Fig. 1b). The observed MHP patterns were consistent throughout our MD simulations. Indeed, despite somewhat blurred MHP “portraits”, similar MHP distributions were obtained as a result of averaging over the ensembles of MD conformations (Supplementary Fig. 2). It is interesting to note that the gates in the π-semiopen-V1 and α-open-V1 states were closed more tightly in unrestrained MD simulations compared to the corresponding cryo-EM structures and their hydrophobicity increased to the values of π-closed-V1 and α-closed-V1, respectively.

**Fig. 3:**
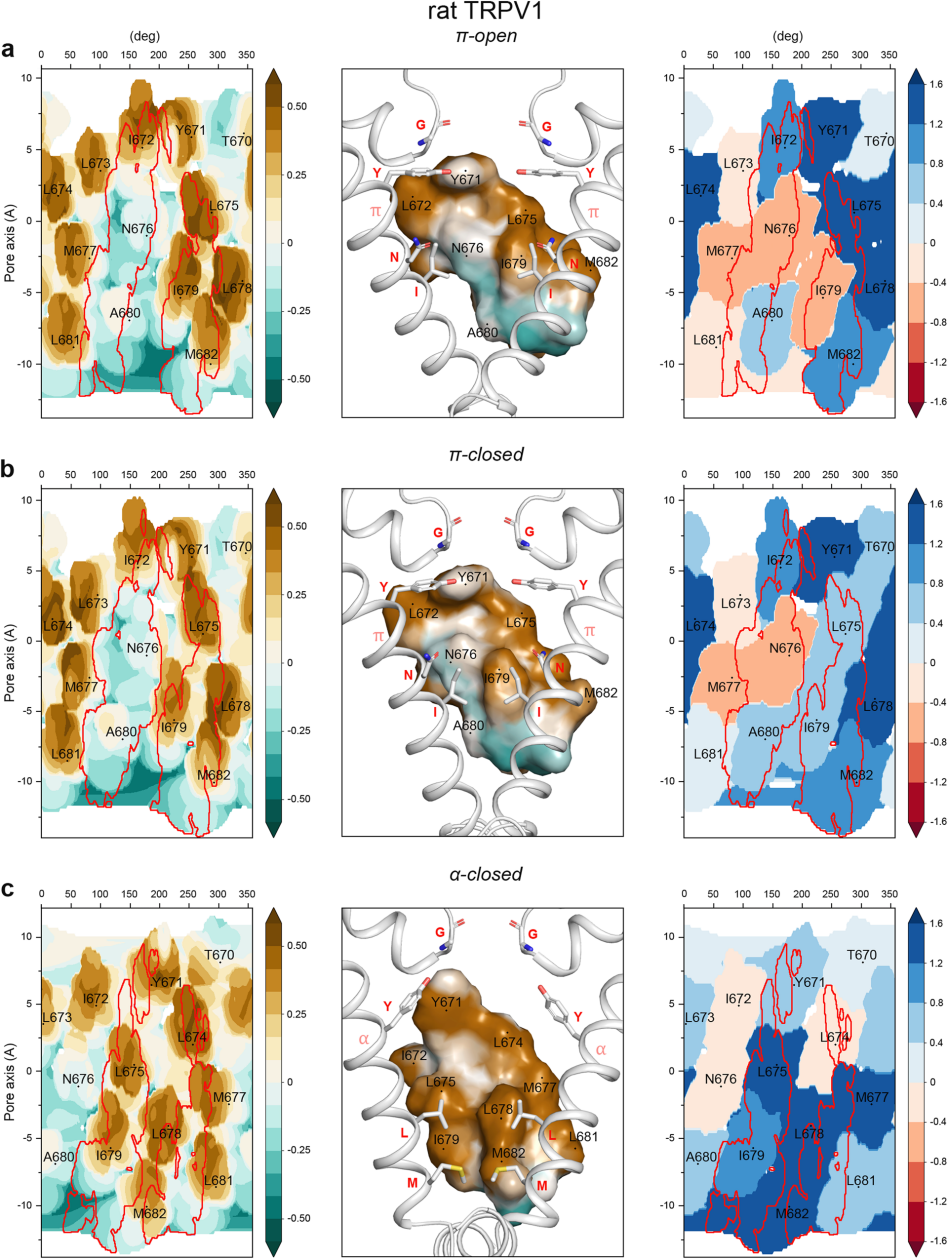
Contact interfaces between S6 helices at the gate region of rat TRPV1. **a** – π-open-V1 state, **b** – π-closed-V1 state, **c** – α-closed-V1 state. The panels in each row from left to right: (1) Cylindrical projection of self MHP distribution over the surface of one S6-helix (residues 670-682). The areas of contact with neighbor S6-helices are marked by red curves, the positions of residues on the helix surface are marked on a map, cryo-EM protein structures listed in Table 1 are used for mapping. (2) Surface of the S6-helix fragment (residues 670-682) colored by its MHP values, the positions of residues on the helix surface are labeled. The helix fragment imposed on the two neighboring S6-helices in a cartoon representation. The pore-lining residues are noted by red letters and shown as sticks. (3) Cylindrical projection of 3D–1D profile scores of S6 helix residues 670-682: accessible surface of each residue is colored according to the score value. The coloring scheme is shown on the right. The areas of residue-residue contacts for neighbor S6 are marked with red contours.

To investigate the evolution of contacts between S6 helices at the gate region during interstate transitions, we mapped the self MHP of one S6 helix on the cylindrical representation of its surface and used this map to highlight the contact areas between this helix and the neighboring S6 helices (left panels in Fig. 3). In π-open-V1, the lower part of S6 contacts its neighbors via two areas. The first, mainly hydrophilic contact area is contributed by I672, N676 and A680, while the second, mainly hydrophobic contact area involves L675, I679, and M682 (left panel in Fig. 3a). Given the 4-fold symmetry of the S6 bundle, the 3D representation of the MHP distribution suggests that in the π-open-V1 state, the hydrophilic surface of the first contact relies on the hydrophobic contact of the neighboring subunit; the same is true for the second hydrophobic contact area (central panel in Fig. 3a). Thus, unfavorable hydrophilic-hydrophobic contacts are formed between the S6 helices in the gate region. Similar contact interfaces were observed in π-closed-V1 and π-semiopen-V1 (Fig. 3b and Supplementary Fig. 3a).

Nonetheless, the energetic preferability of these states is different, which can be illustrated by mapping the packing parameters of S6 using 3D–1D profiles. In this method, the higher scoring function values reflect more favorable environment for a residue, when polar/non-polar properties of this residue match the corresponding properties of the neighboring residues and water. Vice versa, the negative values characterize unfavorable environment (right panels in Fig. 3). In π-open-V1, two residues at the interface show poor packing quality: N676 because of its hydrophobic environment and I679 due to its exposure to water (right panel in Fig. 3a). While π-semiopen-V1 and π-closed-V1 show improved environment quality for I679 residues that are tightly packed at the closed gate, the environment of N676 remains unfavorable (Fig. 3b and Supplementary Fig. 3a).

In α-closed-V1, N676 leaves the contact area, and both contact interfaces between S6 helices become mainly hydrophobic (Fig. 3c). Residues L675, L678, I679, and M682, which form those contact regions, have high quality environment, suggesting that α-closed-V1 is energetically more favorable for the gate than the states with π-bulge in the middle of S6. On the contrary, in α-open-V1 (the state with similar S6 interface topology), the same set of residues has a much less favorable environment, indicating a transitional or intermediate character of this state (Supplementary Fig. 3b).

### TRPV1, TRPV3, and TRPV6 pores have similar hydrophobic organization

**Fig. 4:**
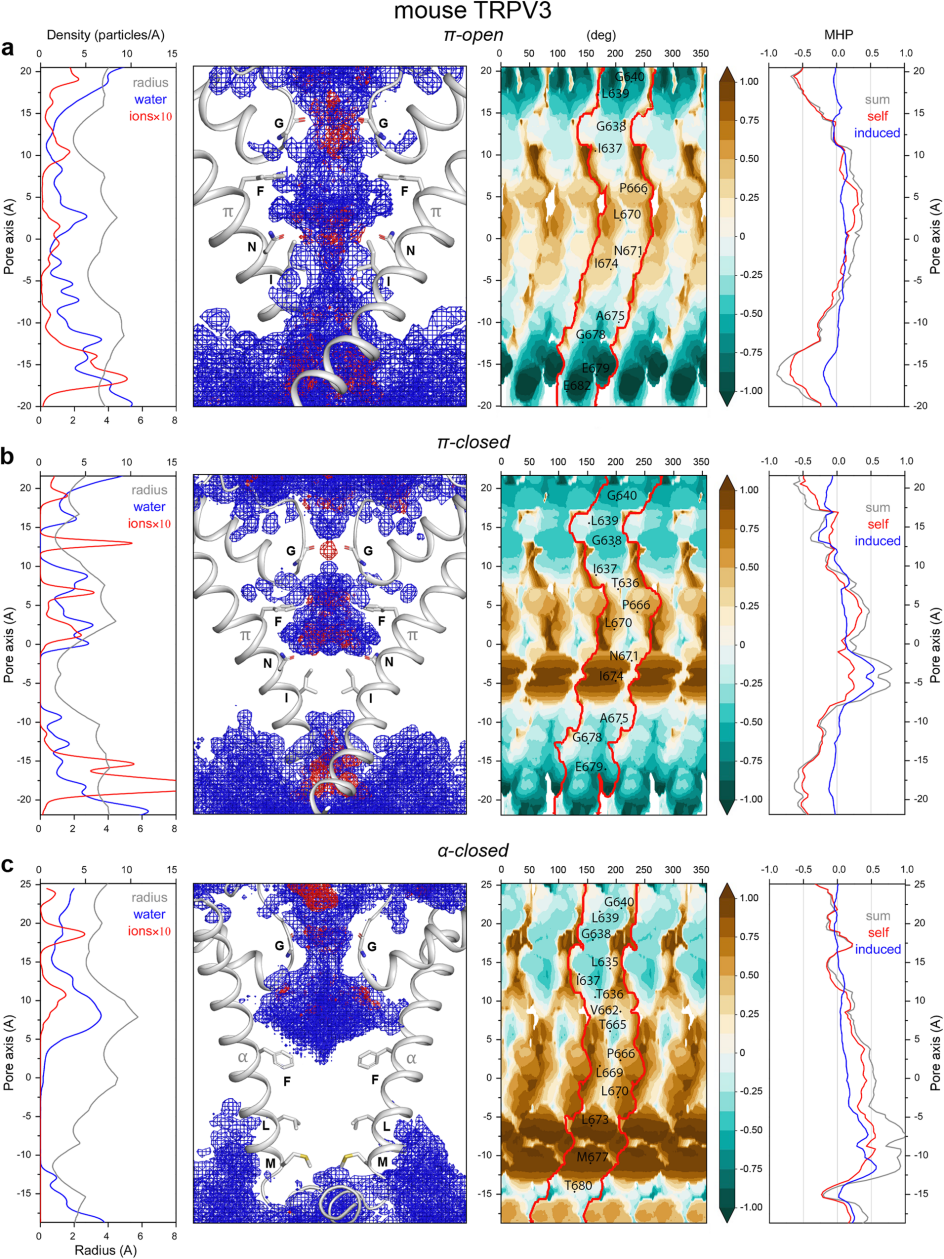
Pore properties of mouse TRPV3. **a** – open state with π-bulge conformation of S6 helix (π-open-V3), **b** – closed state with π-bulge conformation of S6 helix (π-closed-V3), **c** – closed state with α-helical conformation of S6 helix (α-closed-V3). All designations are the same as in Fig. 2.

To sample different states of TRPV3, we selected four structures of mouse TRPV3: three states with π-bulge in the middle of S6 – fully open (π-open-V3), apo closed (π-closed-V3) and sensitized (π-semiopen-V3) – and one with α-helical S6 (α-closed-V3)^15,18^ (Table 1). TRPV3 states show pore properties similar to the corresponding states of TRPV1. The π-open-V3 state is fully hydrated during MD simulations. It has a moderately hydrophobic belt contributed by residues P666, L670, N671, and I674 (Fig. 4a). In π-closed-V3, the pore has two narrow constrictions: formed by (1) G638 at the selectivity filter and (2) tightly packed I674 side chains at the hydrophobic gate. The gate stays dry throughout MD simulations, while the selectivity filter coordinates a partially dehydrated ion (Fig. 4b). The π-semiopen-V3 state is similar to π-closed-V3, but its activation gate is 30% less hydrophobic and not permeable to water or ions (Supplementary Fig. 1c). The highly hydrophobic gate of α-closed-V3 is contributed by side chains of L637 and M677, and the pore stays non-conducting during the entire MD simulation (Fig. 4с). In contrast to α-closed-V1, the selectivity filter in α-closed-V3 is widely open, and two additional S6 residues (T665 and V662) are exposed to the pore. The interhelical contacts at the gate region of TRPV3 also have similar topology to TRPV1. The π-bulge states are characterized by unfavorable hydrophilic-hydrophobic contacts, with poorly packed N671 and I674 in π-open-V3 (Supplementary Fig. 4a) and more tight and preferable packing of the gate residues in π-semiopen-V3 and π-closed-V3 (Supplementary Figs. 3c and 4b). In α-closed-V3, both sides of the contact interfaces are hydrophobic, and the contact residues have high-quality environment (Supplementary Fig. 4c). The poor quality of N671 environment in α-closed-V3 is the result of the hydrophobic environment of the helix S5 contacting with this residue. However, during MD simulations, N671 formed one or two hydrogen bonds with the backbone of S5, which partially compensated for the unfavorable packing of this residue.

**Fig. 5:**
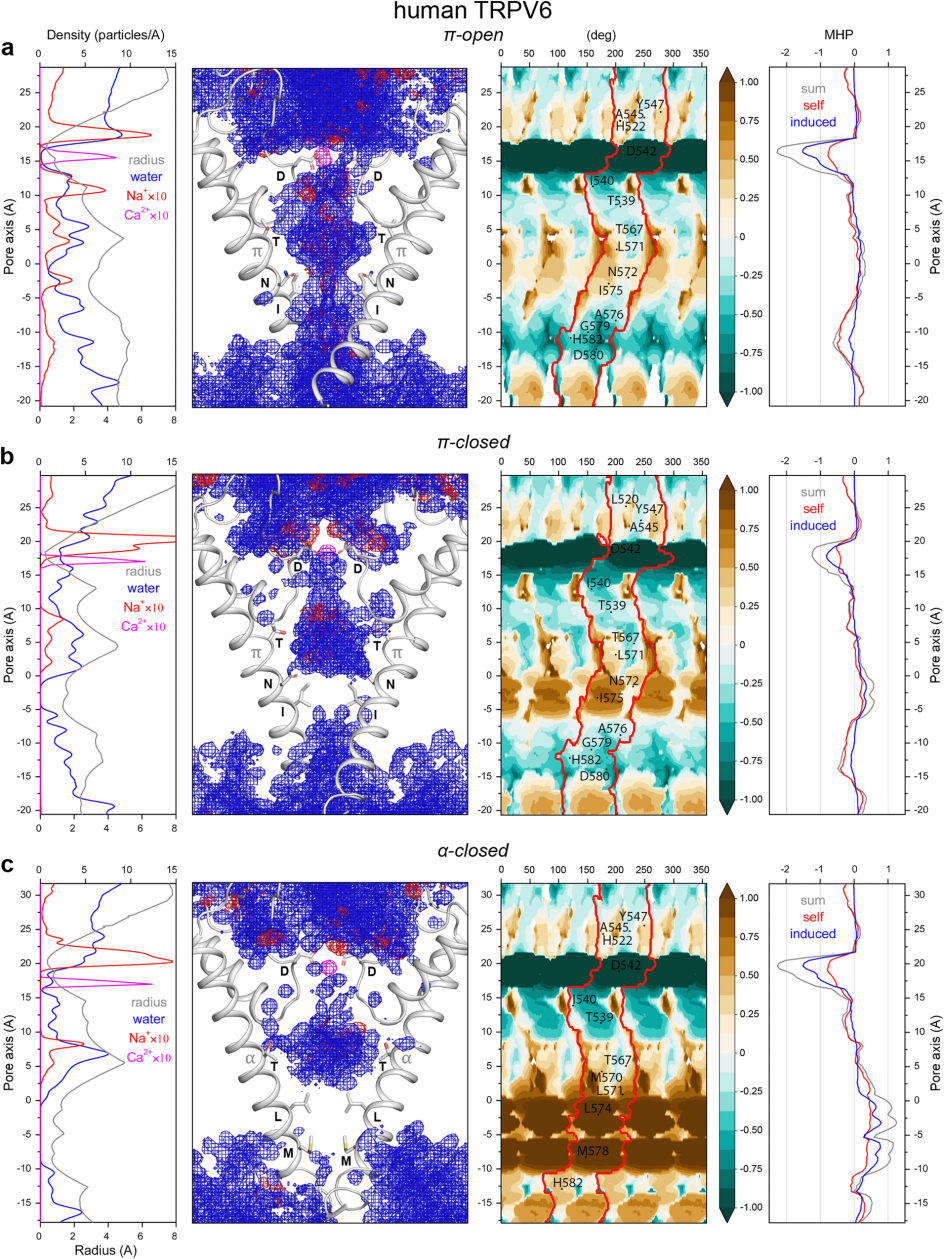
Pore properties of human TRPV6. **a** – open state with π-bulge conformation of S6 helix (π-open-V6), **b** – closed state with π-bulge conformation of S6 helix (π-closed-V6), **c** – closed state with α-helical conformation of S6 helix (α-closed-V6). Density of Ca^2+^ ions averaged over MD simulations is shown with magenta. All other designations are the same as in Fig. 2.

The set of states for human TRPV6 was represented by structures in the open state with π-bulge in the middle of S6 (π-open-V6), the inhibitor econazole-bound closed state with α-helical S6 (α-closed-V6) and the calmodulin-bound inactivated state with π-bulge-containing S6 (π-closed-V6)^19,20^ (Table 1). TRPV6 is somewhat different from TRPV1 and TRPV3, because its selectivity filter is highly selective to Ca^2+^. The selectivity filter residues D542 form a highly hydrophilic narrow constriction that keeps a Ca^2+^ ion bound throughout MD simulations in all TRPV6 states (Fig. 5). Also different, the polar side chains of T539 and T567 (residues I642 and Y671 at the corresponding positions in TRPV1 or I637 and P666 in TRPV3) create much less hydrophobic environment in the central cavity of the pore. Nevertheless, the gate region behavior is similar in all three channels. The π-open-V6 state has a fully hydrated, ion-conducting pore with the narrow hydrophobic region of the S6 bundle lined by residues L571, N572, and I575 (Fig. 5a), similar to TRPV1 and TRPV3 (Figs. 2a and 4a). The π-closed-V6 structure represents a state inhibited by calmodulin, where the gate-forming residues I575 are packed less tightly than in the corresponding π-closed states of TRPV1 and TRPV3. While the gate is less hydrophobic, the π-closed-V6 pore remains non-conductive throughout MD simulations (Fig. 5b), similar to the π-semiopen states of TRPV1 (Supplementary Fig. 1a) and TRPV3 (Supplementary Fig. 1c). The gate region of α-closed-V6 is formed by L574 and M578 (the same residues as in the corresponding states of TRPV1 and TRPV3) and stays dry during MD simulations (Fig. 5c). Contact topology of S6 helices in different TRPV6 states (Supplementary Fig. 5) is also similar to TRPV1 (Fig. 3) and TRPV3 (Supplementary Figs. 3c and 4). The poor packing quality of N572 in α-closed-V is also related with hydrophobic environment of the neighboring S5 helix.

Our analysis identifies three categories of TRPV channel states based on characteristics of their pores. The channel pores in the π-open states are permeable to ions and water, with the gate region showing low hydrophobicity. The π-closed and π-semiopen states have non-permeable pores with the hydrophobic gate formed by tightly packed isoleucine side chains. The α-closed and α-open states are also non-conducting, but the hydrophobic gate is formed by the tightly packed side chains of leucine and methionine residues. The transition π-open→π-semiopen→π-closed is accompanied by the formation of a hydrophobic constriction in the TRPV channel pore. The increase in hydrophobicity is not mainly caused by exposing additional hydrophobic residues to the pore, but by more tight packing of the isoleucine side chains. This phenomenon is reflected in the MHP profiles as a substantial contribution of the induced MHP. In the α-open and α-closed states, the S6 transition to a fully α-helical conformation results in exposure to the pore of non-polar leucine and methionine residues that form more extended hydrophobic gates.

**Fig. 6:**
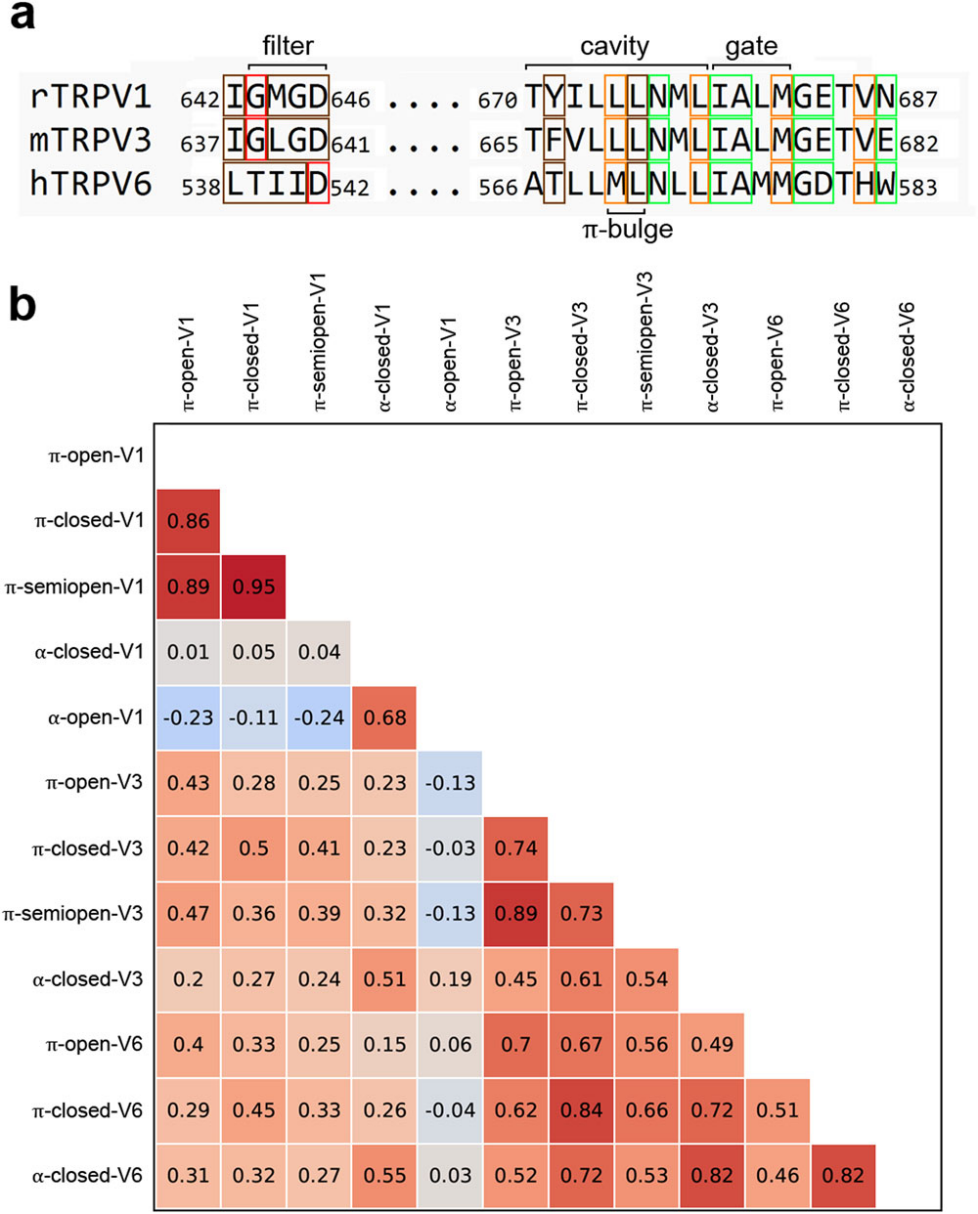
Structural alignment and pairwise correlation coefficients of 3D–1D profile scores of the TRPVs pore regions. **a** – structure-based sequence alignment of the pore regions in rat TRPV1, mouse TRPV3 and human TRPV6. Green frames indicate residues exposed to the pore when S6 contains the π-bulge, orange frames indicate residues exposed to the pore when S6 is α-helical, brown frames indicate residues lining the pore in all states, red frames indicate residues forming the “bottle neck” of the filter. Residues contributing to the filter, central cavity, gate, and π-bulge are labeled. **b** – The diagonal matrix of pairwise correlation coefficients for 3D–1D profile scores of S6 helices calculated between TRPV states from Table 1 (used 3D–1D profiles of 670-687 residues in the sequence numeration of rat TRPV1). Heatmap corresponds to the correlation coefficient values.

To further compare the states of TRPV channels, we calculated the pairwise correlation coefficients (R) for their 3D–1D profiles along the aligned sequences of S6 helices (Fig. 6). Such analysis revealed both common and specific properties of these channels. The π-open, π-semiopen, and π-closed states of TRPV1 and TRPV3 group into highly correlated clusters (*R* > 0.7) that differ from α-closed and α-open (*R* ≤ 0.6). Such a difference is especially pronounced in the case of TRPV1. Conversely, the highly correlated states of TRPV6 are π-closed and α-closed (*R* = 0.8), both different from π-open (*R* ≤ 0.5). Correlations between the 3D–1D score profiles for the three channel subtypes show that TRPV1 and TRPV6 are most different and TRPV3 has intermediate properties. The fact that the correlated cluster distributions vary between different TRPV channel subtypes indicates that 3D–1D profiles are not directly related to the secondary structure of S6 (α-helix or π-bulge) but rather depend on the spatial arrangement of residues in the pore region.

### The open pore is more hydrophobic than the closed one in TRPV1 and TRPV3 but not in TRPV6

One of the putative mechanisms of TRPV channel thermosensitivity is a positive difference in heat capacity between the open and closed conformations that may arise from changes in exposure of residue side chains to water as a result of channel opening. Clapham and Miller estimated that exposure of 300 methylene groups or aromatic carbons to water should be sufficient to generate strong temperature dependence of TRP channels^27^. To find the probable hydrophobic thermosensor in the channel pore we compared MHP distributions over the water-exposed pore surface between the selectivity filter and activation gate of different TRPV states.

**Fig. 7:**
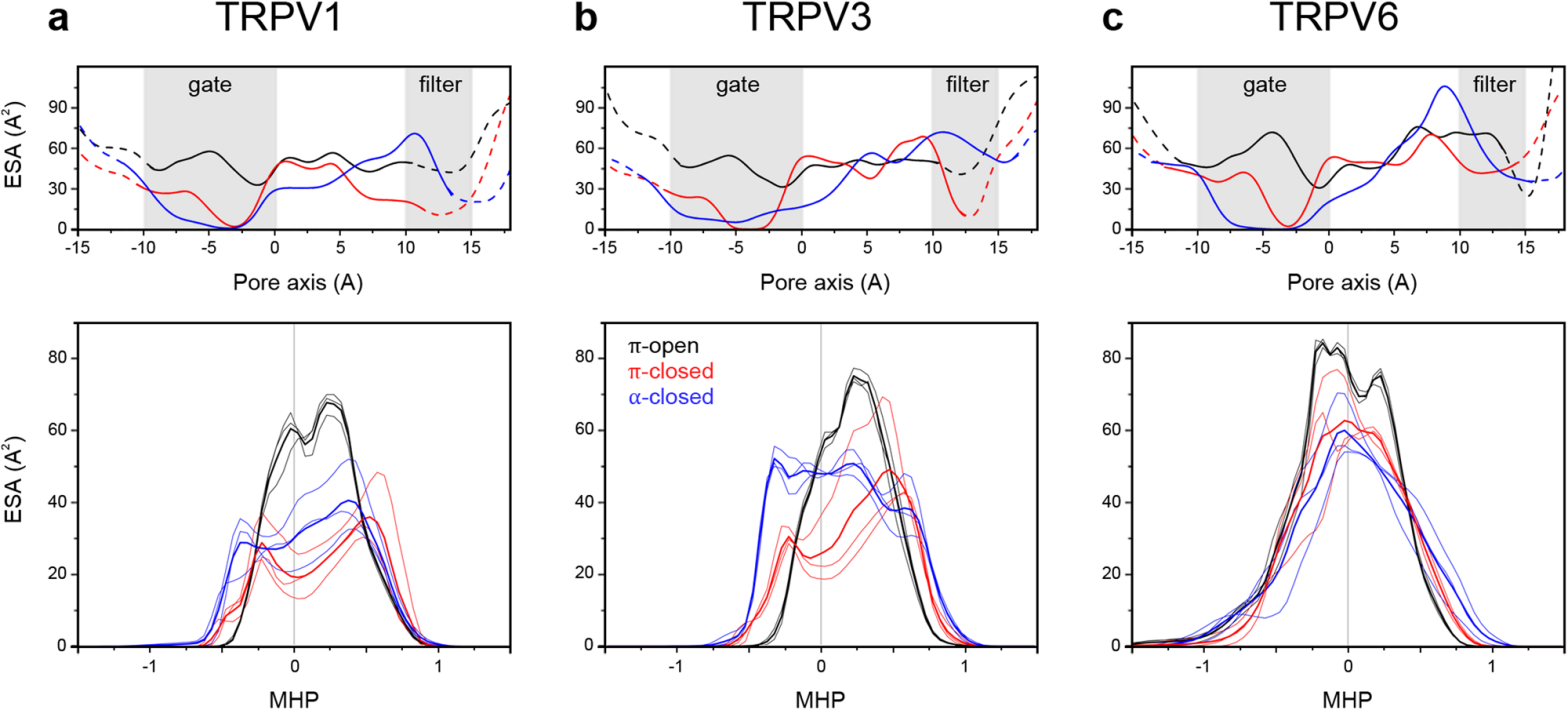
Cumulative surface properties of the TRPVs pore regions. Water-exposed protein surface area (ESA) along the pore axis (top panels) and the distributions of ESA depending on its MHP value (lower panels) for rat TRPV1 (**a**), mouse TRPV3 (**b**), and human TRPV6 (**c**). Black curves represent π-open states, red π-closed states, and blue α-closed states. Thin lines represent data from three independent MD simulations, thick lines represent an average of the three. Shaded gray areas in the top panels labels the selectivity filter and gate regions of the pore. In the lower panels, MHP values > 0 show hydrophobic and MHP < 0 hydrophilic exposed surface area.

The π-closed-V1 → π-open-V1 transition is indeed accompanied by an increase in the water-exposed (or hydrated) surface area (ESA), both total (ΔESA_T_ = 360 ± 30 Å^2^) and hydrophobic (ΔESA_H_ = 260 ± 30 Å^2^). The latter increase in the surface area is mainly due to the appearance of hydrophobic residues I679 and A680 at the gate surface (Fig. 7a). A total of 16 additional methylene groups were exposed to water, which is in good agreement with our previous estimates^24^. Because of the high variability in hydration of the closed pore during MD simulations, our calculations do not provide an accurate estimation of ΔESA during the α-closed-V1 → π-closed-V1 transition. However, we can conclude that such a transition is accompanied by some decrease in the hydrated area: ΔESA_T_ = −170 ± 50 Å^2^ and ΔESA_H_ = −74 ± 40  Å^2^. The hydrated surface is redistributed from the extracellular part of the pore in the α-closed state to the gate region in the π-closed state. In TRPV3, the π-closed-V3 → π-open-V3 transition shows a similar trend with respect to the total and hydrophobic water-exposed surface of the pore: ΔESA_T_ = 150 ± 50 and ΔESA_H_ = 180 ± 40 Å^2^ (Fig. 7b). However, the α-closed-V3 → π-closed-V3 transition is accompanied by a noticeable decrease in the total (ΔESA_T_ = −330 ± 50 Å^2^) and hydrophobic surface area (ΔESA_H_ = −100 ± 50 Å^2^), which is manly related to the wide opening of the TRPV3 selectivity filter in α-closed-V3 (Fig. 4c). During the π-closed-V6 → π-open-V6 transition in TRPV6, the increase in the hydrated hydrophobic area is much less pronounced than in TRPV1 and TRPV3 (ΔESA_H_ = 40 ± 10 Å^2^). Nevertheless, the total exposed area increased by about 230 ± 40 Å^2^ (Fig. 7c). The α-closed-V6 → π-closed-V6 transition leads to redistribution of the hydrated area from the gate region to the upper part of the pore without changing the hydrated area: ΔESA_T_ = 20 ± 50 and ΔESA_H_ = −5 ± 20 Å^2^.

We conclude that the exposure of additional hydrophobic groups to water takes place in the pores of thermosensitive channels TRPV1 and TRPV3, while almost entirely absent in non-thermosensitive TRPV6. However, this effect is an order of magnitude weaker than required for temperature activation: ~100–300 Å^2^ of exposed hydrophobic area or a dozen of methylene groups instead of hundreds of methylene groups proposed by Clapham and Miller^27^. It therefore appears that the hydrophobic pore of TRPV channels unlikely represents a key part of their thermosensor. However, changes in the hydrated hydrophobic surface of the pore may fine tune TRPV gating and are likely related to specific functional characteristics of different channels subtypes.

## Discussion

In this study we performed a detailed computational analysis of multiple gating intermediates for three TRPV channels (Table 1). Only three common categories of conserved pore states were identified: π-open, π-closed, and α-closed. There are other structures to consider but they seem to represent intermediate or transitional states between the main three. We classified the pore states based on the following set of criteria: (1) conformation of the pore-forming S6 (α-helical or with the π-bulge in the middle), (2) hydration and ion conductivity of the pore during MD simulations, (3) hydrophobicity portrait of the gate, (4) quality of residue packing at the gate region, and (5) complementarity of hydrophilic/hydrophobic properties of contacts between S6 segments of the gate.

The proposed classification of TRPV channel pore states fully agrees with the earlier described model of TRPV3 gating^15,28–30^, which follows the α-closed→π-closed→π-open transition. We show that the most energetically preferable and, as a result, the most structurally stable conformation of the TRPV channel gate is the closed state with entirely α-helical S6 (α-closed). This state is characterized by the highest quality of non-polar residue packing at the gate and the highest interhelical contact complementarity between the S6 segments. The gate in the α-closed state is formed by the tightly packed leucine and methionine residues that form an extended and highly hydrophobic constriction of the pore, which is not permeable to water or ions. The peculiarity of this state is a very dense and favorable packing of non-polar residues at the lower gate, which is reflected in high values of the 3D–1D score and induced MHP (see the parameters of the helix-helix interfaces in Fig. 2c and Supplementary Figs. 4c and 5c and profiles in the right panels in Figs. 1c, 3c, and 4c, respectively). In addition, compared to the other states, this state is characterized by a substantially increased size of the cavity above the lower gate, filled with water and ions, as becomes apparent from the pore radius profiles (Figs. 1c, 3c, and 4c, left panel). Accordingly, the α-closed state has the smallest contact area between the neighboring protein fragments, even despite the strong hydrophobic gate. A similar hydrophobic patch composed of non-polar groups of valine, alanine, and threonine was shown to be responsible for the stability of the closed conformation of prokaryotic potassium channel KcsA^31^.

The key feature of TRPV channel activation is structural transformation of the gate region as a result of the α-to-π transition in S6, which leads to the rotation of the lower part of S6 by about 100°. After this first step of activation, the gate is formed by isoleucine residues, the hydrophobic constriction is less extended, and the preferable hydrophobic-hydrophobic contact between S6 segments changes to the less stable hydrophilic-hydrophobic contact. The resulting π-closed state remains non-permeable, but it has highly modified geometry and MHP portrait of the pore gate compared to α-closed. Further transition to the π-open state is a consequence of S6 segments moving apart and releasing the hydrophobic constriction of the pore. This transition occurs without substantial conformational changes in the pore domain. Accordingly, the gate region of S6 serves as a hydrophobic conformational trigger, which provides a hopping transition from the energetically stable α-closed state to the less favorable π-closed state and further to the unstable π-open state. Interestingly, the gating trigger described above is not the only one found in TRPV channels. Lipid molecules bound to the surface of TRPV3 in the closed state were shown to dissociate from the channel and act as a gating trigger upon channel opening^15,18,28^. Thus, the TRPV channel function is regulated by at least two “fuses” – the protein itself and the annular lipids. It is important to note that the two fuses are close in space and can act in concert during channel activation. Perhaps, this joint operation of the molecular “switches” is critical for multifaceted regulation of TRPV channels, including their activation by temperature and a variety of ligands. A better understanding of this operation will uncover new strategies to regulate the function of this important class of ion channels.

The π-bulge in S6 is a conserved structural feature of TRPV channels^10,11,16,29,32^ that seems to be a critical element of channel gating. The rotation of S6 that turns the methionine out of the pore seems to be a necessary precursor of channel opening^5^. Thus, in TRPV3 channel that require repeated or prolonged exposure to activating stimuli in order to reach full activation, the α-closed→π-closed transition may signify channel sensitization. Otherwise, TRPV channels may convert to the α-closed inactivated state after binding of either agonists or antagonists, e.g., capsaicin to TRPV1^33^, cannabidiol to TRPV2^34^, HC-067047 to TRPV4^32^ or econazole to TRPV6^20^. Under physiological conditions, TRPV5 and TRPV6 channels are constitutively open in the apo condition, so the energy preference towards the π-open state may be provided by structural elements outside the hydrophobic gate.

Other TRPV subtypes exhibit the same categories of states. Apo, RTX- and CBD-bound structures of rat TRPV2 represent conformations with entirely α-helical S6 and the gate formed by L641 and M645^34,35^. Meanwhile, there is another apo conformation of a wild-type rat TRPV2 with the *π-open* conformation of the gates formed by I642 and wide enough to provide fully hydrated pore^36^ (PDB ID 6BO4). Mouse TRPV2 with bound cholesterol and 2-APB are the π-closed states with the gate formed by I637^37^. Human TRPV4 structures demonstrate all three states: agonist-bound π-open, apo π-closed, and antagonist-bound α-closed with the gate composed of I715 or L714 and M718, respectively ^32,38^. Apo-state structures of rabbit TRPV5 captured at different pH represent the π-closed state with different extents of pore occlusion at the gate composed of I575^39^. The same channel bound to PIP_2_ lipid at pH ≥ 6 represents the π-open state^39,40^. The calmodulin-bound structure of TRPV5 looks similar to π-closed TRPV6 with I575 forming the gate^40,41^. A few exotic states of TRPVshave been reported recently. Pentameric human TRPV3 with α-helical conformation of S6 and pore-oriented L673 and M677 is a wide-open channel due to an excessive number of monomers^6^. Human TRPV6 inhibited by genistein represents a mixed closed state with two oppositely paced S6 in α-helical conformation and two others with the π-bulge^42^.

Zhang et al.^14^ reported a structure of rat TRPV1 in complex with the DkTx toxin as an open state with entirely α-helical S6 and wide lumen of its hydrophobic gate (*R*_gate_ = 1.2 Å, PDB ID: 7L2U). Some rat TRPV2 structures (e.g., PDB IDs: 7N0N and 7T37) that appeared to be similar to this α-open-V1 structure were interpreted as intermediates on the path to opening^35^ (*R*_gate_ = 1.9 Å). We propose that these representatives of the α-open states are non-conductive. This proposition agrees with the free energy calculations, which showed that the dehydrated state is energetically preferable for homogeneous hydrophobic pores with even less constricting dimensions (radius < 7.5 Å and height 16 Å)^43^. The states with π-bulge in the middle of S6 seem to be much more suitable for pore conductance, represented by numerous cryo-EM structures with the open gate (*R*_gate_ ≈ 2.9 Å) and pores filled with water and ions throughout our MD simulations of the π-open states. In all these structures, the hydrophilic asparagine places its side chain oxygen near the hydrophobic gate. Our earlier analysis showed that this oxygen atom may contribute to hydration of the open TRPV1 pore by localizing up to 6 waters near the gate and substantially reducing the energy barrier for ion transport^44^. The importance of N676 for the TRPV1 channel function as a facilitator of pore hydration has also been discussed in other MD studies^45^. Our recent work showed that the oxygen atom of asparagine can coordinate water molecules in such a way that it restores the hydration shell of ions at the hydrophobic gate of TRPV1, thus facilitating ion transport through the gate in a fully hydrated state^46^. There are no such water coordinating groups near the gate when S6 is entirely α-helical.

The study of human TRPV6 using electrophysiology^13^ showed that point mutations of A616 and M617 (A576 and M577 in our numbering) to hydrophilic residues resulted in increased Ca^2+^ conductance but had little effect on current carried by monovalent ions. To explain this phenomenon, the authors proposed a “double residue gate” mechanism for TRPV5 and TRPV6. However, these residues are different from the gate-forming residues in the α-closed (L574 and M578) or π-closed (I575) states. Therefore, based on structural data and our computational analysis, we suggest that A576 and M577 cannot form the gate or lead to any substantial instability of interhelical contacts at the gate region. Most probably, the observed functional effects can be explained by reduced Ca^2+^ inactivation of the mutant channels.

We would like to emphasize that the activation mechanism we propose for TRPVs is not common among other ion channels. For example, the available structures of potassium channels hKca3.1 and KcsA show that their activation is caused by simple translation of the pore-forming helices away from the pore axis^47,48^. Another example is ionotropic glutamate receptors (iGluRs) – tetrameric ion channels, which opening occurs as a result of kinking the pore-lining helices M3 (aka S6), very different from TRPV channels^49^! During these conformational changes, the change in characteristics (2)-(5) described above occurs without trigger switching (like α to π-helix transitions) or helix packing disturbances. Accordingly, we propose that the combination of criteria (1)-(5) forms a specific “dynamic molecular portrait” of the pore domain that shows fine nuances of its hydrophobic organization and structural flexibility. As the number and variability of available TRPV structures increases, the DMP analysis will provide deeper understanding of the gating mechanism and help to reveal structural features responsible for functional differences between channel subtypes. Other families of ion channels also possess hydrophobic gates^7,31,50–52^, and it would be useful to study their hierarchy of gating transitions using the approach proposed in this study. This may provide valuable information about the ion channel structure and function, complementary to structural information from PDB models.

## Methods

### MD simulations

The list of TRPV structural models used in the study is shown in Table 1. Missing fragments of the loops in the models were reconstructed using the RCS+ server^53^. If necessary, missing side chains of residues were added using the PDB2PQR server^54^. Prepared TRPV models were inserted into a hydrated lipid bilayer with the molecular composition of 50% palmitoyloleoylphosphatidylcholine (POPC), 25% palmitoyloleoylphosphatidylethanolamine (POPE), and 25% cholesterol (about 900 molecules in the membrane). Na^+^ and Cl^-^ ions were added to ensure zero net charge at 0.15 M ionic concentration. One Na^+^ was placed in the central cavity of the pore of closed channels to increase their hydration rate. In the case of TRPV6, Ca^2+^ were inserted in the selectivity filter like in the crystal or cryo-EM structures.

The simulated systems were first equilibrated in several stages: 5×10^3^ steps of steepest descent minimization followed by heating from 5 to 310 K during a 100 ps MD run, then 10 ns of MD run with fixed positions of the protein heavy atoms and ions, 10 ns of MD with fixed positions of the protein backbone and ions, 10 ns of MD with fixed positions of the protein C_α_ atoms and ions, 10 ns of MD with fixed positions of the protein C_α_ atoms only (in total, 40 ns equilibration was performed to provide the pore hydration). Then 200 ns production runs were carried out for each system. For each channel type, three independent trajectories were calculated for the π-open, π-closed, and α-closed states and only one for the other states listed in Table 1. In order to prevent the gate closure in the π-open-V6 state, the distances between the C_α_ atom of residue *i* and the N atom of residue *i* + *4*-were fixed for residues 476-498 of the helix S5. Such constraints did not affect the mobility of the residues forming the gate region. Other systems were modeled without imposing constraints on the proteins.

MD calculations were performed using GROMACS 2021.4 package^55^, Amber99sd-ildn force field^56^ and the TIP3P water model^57^. Simulations were carried out with an integration time of 2 fs, constrained hydrogen-containing bond lengths by the LINCS algorithm^58^, imposed 3D periodic boundary conditions, constant temperature (310 K) and pressure (1 bar). Cutoff distance of 15 Å was used for nonbonded interactions and the particle-mesh Ewald method^59^ used for long-range electrostatics. The modified Lennard-Jones parameters for Na^+^ and Cl^−^ developed by Joung and Cheatham^60^ and for Ca^2+^ developed by Li at all^61^ were used because of their better optimization for modeling of ion-water interaction. The actual values of σ and ε for Na^+^ were 0.2439 nm and 0.3658 kJ mol^−1^, respectively, for Cl^-^ were 0.4478 nm and 0.1489 kJ mol^−1^, for Ca^2+^ were 0.2865 nm and 0.3489 kJ mol^−1^.

### MHP maps and distributions

Molecular Hydrophobicity Potential (MHP) is an empirical quantity to characterize hydrophobic/hydrophilic properties of molecular systems^22^. This formalism is based on the empirical atomic hydrophobicity constants obtained for numerous chemical compounds from the experimentally measured water/n-octanol partition coefficients (P) in the form of logP^22,23,62^. In this study, the MHP value in a molecular surface point is calculated as a superposition of the attenuating contributions *f*_i_⨉exp(−*r*_*ij*_/2), where *r*_*ij*_ is the distance from the surface point *j* to the *i*-th atom and *f*_*i*_ is the hydrophobicity constant of *i*-th atom. Only atoms located at a distance less than *r*_cut_ = 9 Å from the surface point *j* were taken into account. The atomic hydrophobicity constants were obtained using the Wildman and Cripen method^63^.

We used the following algorithm to make cylindrical projection of the MHP distribution on the Van der Waals (VdW) surface of a protein molecule. (1) The pore axis was specified as a spline line passing through the centers of mass of four C_α_ atoms of the same residues in different subunits. The residues 642, 643, 644, 645, 671, 675, 676, 679, 680, 683, and 686 define the pore axis of rat TRPV1 and the residues at the same positions for the other channel subtypes. In all cases, the line is nearly straight and parallel to the membrane plane normal. (2) A set of projection rays was generated. The starting point of each ray lies on the pore axis, ray direction is orthogonal to the axis, each ray going through a node belonging to the regular rectilinear grid on the cylindrical surface of projection with the axis described above and the radius large enough to hold protein atoms inside the surface in any given section along the axis. (3) Each ray was traced until its nearest intersection with the molecular surface. (4) MHP was calculated at each intersection point and the value was assigned to the corresponding grid node on the cylindrical surface. (5) Collected MHP data was plotted as a 2D map representing cylindrical projection of molecular surface MHP distribution.

Ray-tracing loop iterations are independent, allowing trivial workload distribution between multiple CPU cores if available. The search for the ray-surface intersection points is further accelerated by employing the 3ddda algorithm^64^ and consists of two steps. Initially, each voxel in the 3D grid encompassing the whole molecular surface was assigned a list of VdW spheres that may intersect with a ray passing through it. Next, the tracing along each ray occurs until the nearest intersection with the VdW sphere of an atom is found or a ray leaves the grid. During the trace only the voxels the ray going through are considered. And the voxels checked are ordered along the ray length permitting trace stop as soon as a closest point of intersection in the current voxel is found. The described algorithm is one of the variants of the ray tracing method^65,66^ and the grid searching is a fairly effective approach because molecular systems usually do not contain drastic variations of the atoms concentration in space.

The cylindrical projection of the MHP distribution over single S6-helix was calculated employing the same algorithm, but the cylinder axis was a straight line passing through the center of mass of the helix and directed along the principal axis of the inertia tensor corresponding to its largest eigen value. Only C_α_ atoms of the helix were taken into account during inertia tensor computation. Contact zones with the neighboring S6-helices of the helix surface consisted of all points at a distance <7 Å from atoms of 654–692 residues of neighbor subunits for TRPV1 and the residues in similar positions for other channel subtypes. The Molecular Surface Topography tool available at https://model.nmr.ru/cell was used for calculating and plotting the maps.

MHP distributions over the pore surface exposed to water were calculated over the last 50 ns of MD trajectories, when the pores were saturated by water molecules. Here, we used the following algorithm. (1) The point representation of the Connolly surface^67^ was calculated along with the effective area per point. (2) The points within 1.4 Å distance from an oxygen atom of any water molecule located between the filter and activation gates (G643-M682 for TRPV1, G638-M677 for TRPV3, I540-M578 for TRPV6) were selected in each frame. (3) MHP values were calculated for the selected points. (4) Water-exposed molecular surface area along the pore axis and the distribution of exposed surface area depending on its MHP value were calculated and averaged over the MD trajectory. The errors in determining the exposed surface area were calculated as a standard deviation from their average over three independent trajectories.

### 3D–1D profile score distributions

Packing quality in complexes on the basis of 3D profiles was assessed using VERYFY3D^25^. Environmental classes of residues were defined as described by Bowie et al.^26^. The cylindrical projections of 3D–1D profile scores of residues were plotted analogous to the procedure described above for MHP.

### Other analysis

Spatial distributions of water molecules and ions were calculated as their densities averaged over the last 50 ns of MD trajectory and shown as isosurfaces. Density profiles were obtained from the spatial distributions. Profiles of the pore radius and the minimum radius of the gate in Table 1 were calculated with the Hole2 programme^68^. Molecular graphics were rendered using PyMOL v. 2.5.0 (Schrödinger, Inc., New York, NY, USA).

### Reporting summary

Further information on research design is available in the Nature Portfolio Reporting Summary linked to this article.

### Supplementary information

Supplementary Information

Description of Additional Supplementary Files

Supplementary Data 1

Supplementary Data 2

Reporting Summary

## Data Availability

All data needed to evaluate the conclusions of the study are present in the paper or in the Supplementary Information. The initial and final configurations of the systems in MD simulations are available as Supplementary Data 1. Numerical source data for graphs are available as Supplementary Data 2. All other data are available from the corresponding author upon request.

## Code availability

Software needed to evaluate the conclusions of the study are freely available at https://model.nmr.ru/cell.
