# Supplementary material for: Dynamic molecular portraits of ion-conducting pores characterize functional states of TRPV channels
Source: Commun Chem. 2024 Jun 1;7:119. doi: 10.1038/s42004-024-01198-z (PMC11144267; doi:10.1038/s42004-024-01198-z)
Supplement: Supplementary file 1 — Supplementary Information [file 42004_2024_1198_MOESM1_ESM.pdf]

Supplementary Information

Dynamic molecular portraits of ion-conducting pores characterize functional states of TRPV channels

Yury A. Trofimov[1], Nikolay A. Krylov[1], Alexander S. Minakov[2], Kirill D. Nadezhdin[3], Arthur Neuberger[3], Alexander I. Sobolevsky[3], and Roman G. Efremov[1,*]

[1] Shemyakin-Ovchinnikov Institute of Bioorganic Chemistry, Russian Academy of Sciences, 117997, 16/10 Miklukho-Maklaya st., Moscow, Russia

[2] M.V. Lomonosov Moscow State University, 119991, Leninskie Gory, Moscow, Russia

[3] Department of Biochemistry and Molecular Biophysics, Columbia University, NY 10032, 701 West 168th Street, New York, USA

* Corresponding author. E-mail: efremov@nmr.ru; Tel.: +7-903-743-16-56

This PDF file includes:

Supplementary Figures 1-5

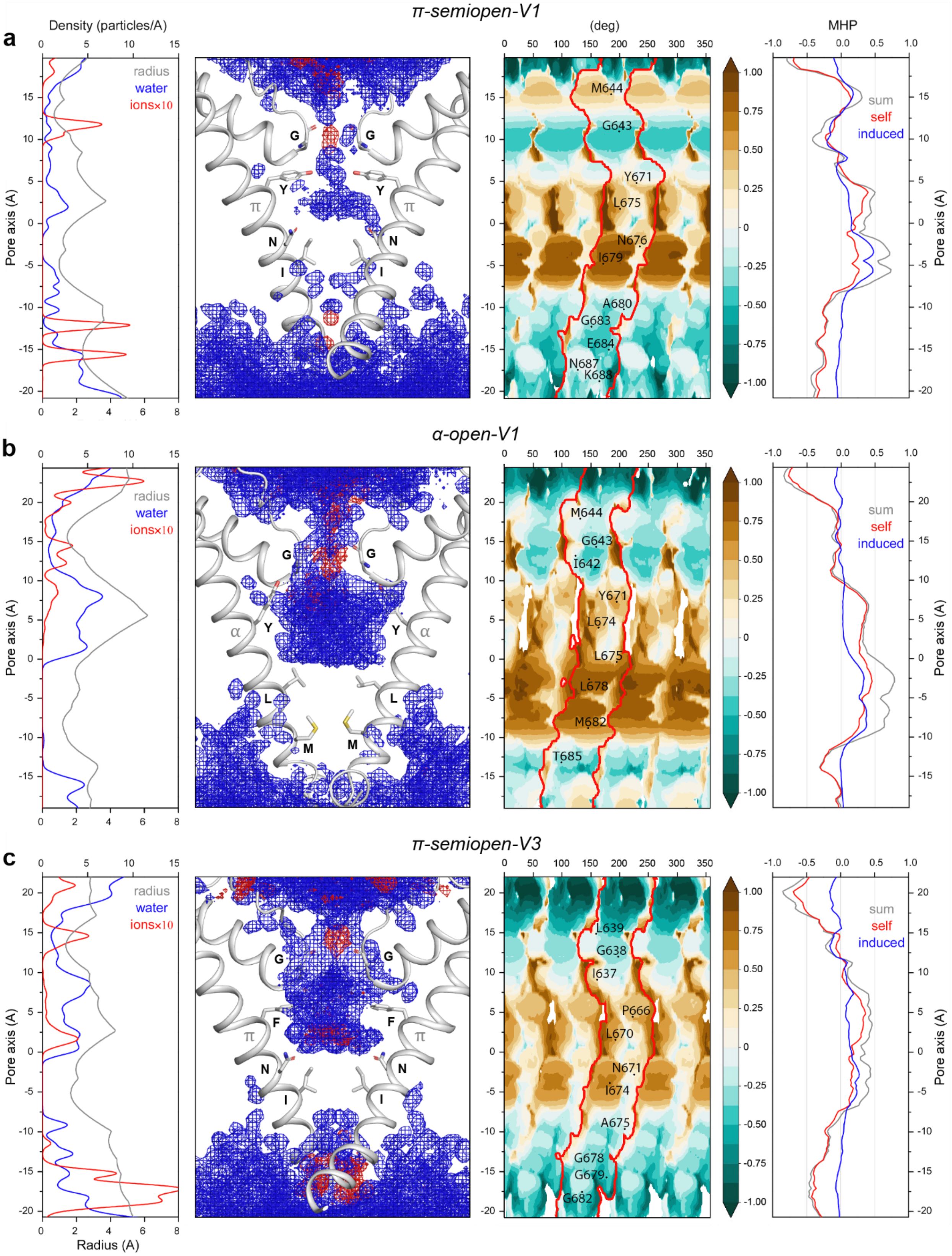

Supplementary Fig. 1 | Pore properties of rat TRPV1 and mouse TRPV3. a – semiopen state of TRPV1 with π-bulge conformation of S6 helix (π-semiopen-V1), **b** – open state of TRPV1 with α-helical conformation of S6 helix (α-open-V1), **c** – semiopen state of TRPV3 with π-bulge conformation of S6 helix (π-semiopen-V3). All designations are the same as in Fig.2.

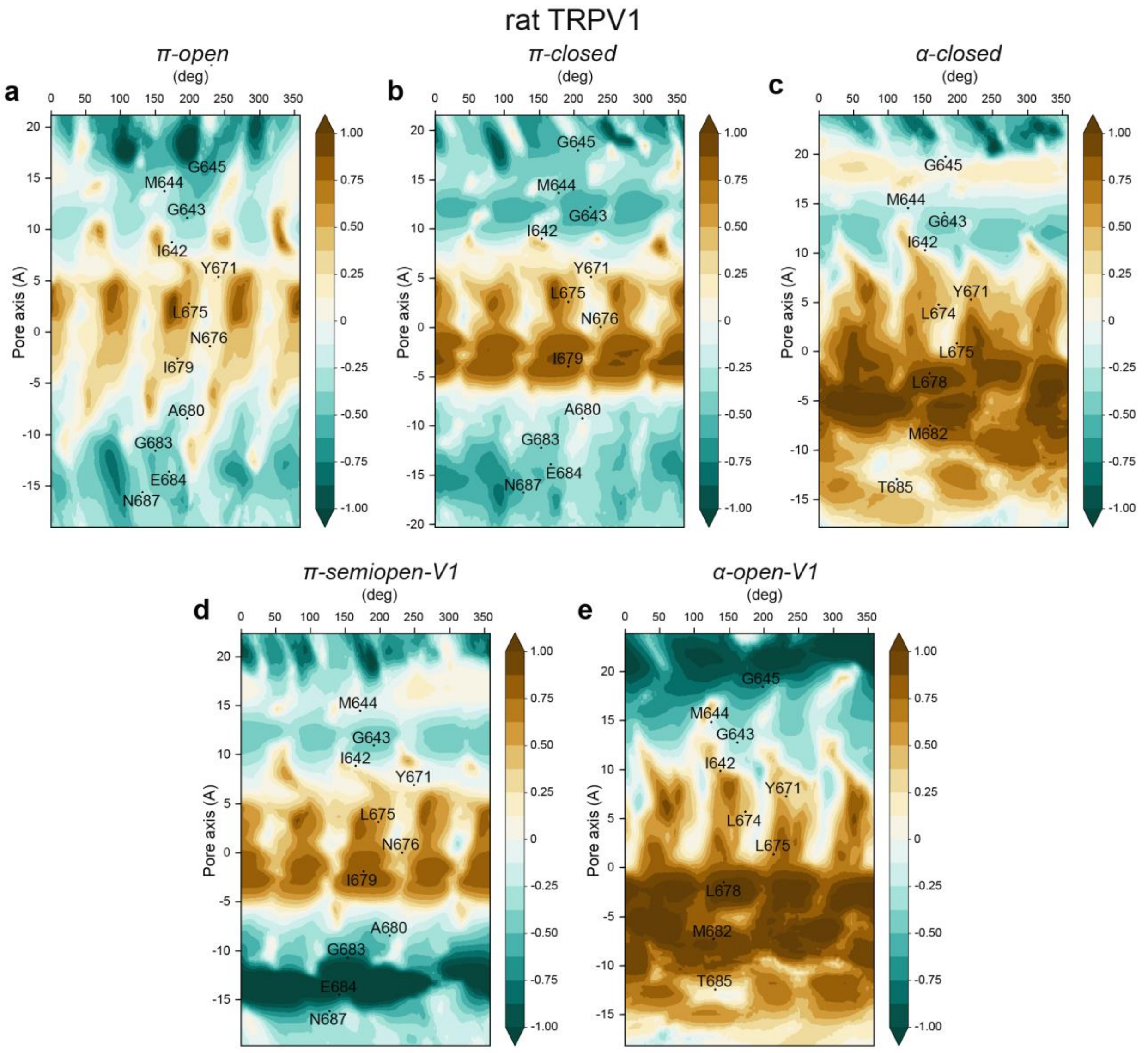

Supplementary Fig. 2 | Pore MHP maps of rat TRPV1 calculated as an average over the last 50 ns of MD simulations. a – open state with π-bulge conformation of S6 helix (π-open-V1), **b** – closed state with π-bulge conformation of S6 helix (π-closed-V1), **c** – closed state with α-helical conformation of S6 helix (α-closed-V1), **d** – semiopen state with π-bulge conformation of S6 helix (π-semiopen-V1), **e** – open state of TRPV1 with α-helical conformation of S6 helix (α-open-V1). Color scheme and marks are the same as in Fig.2.

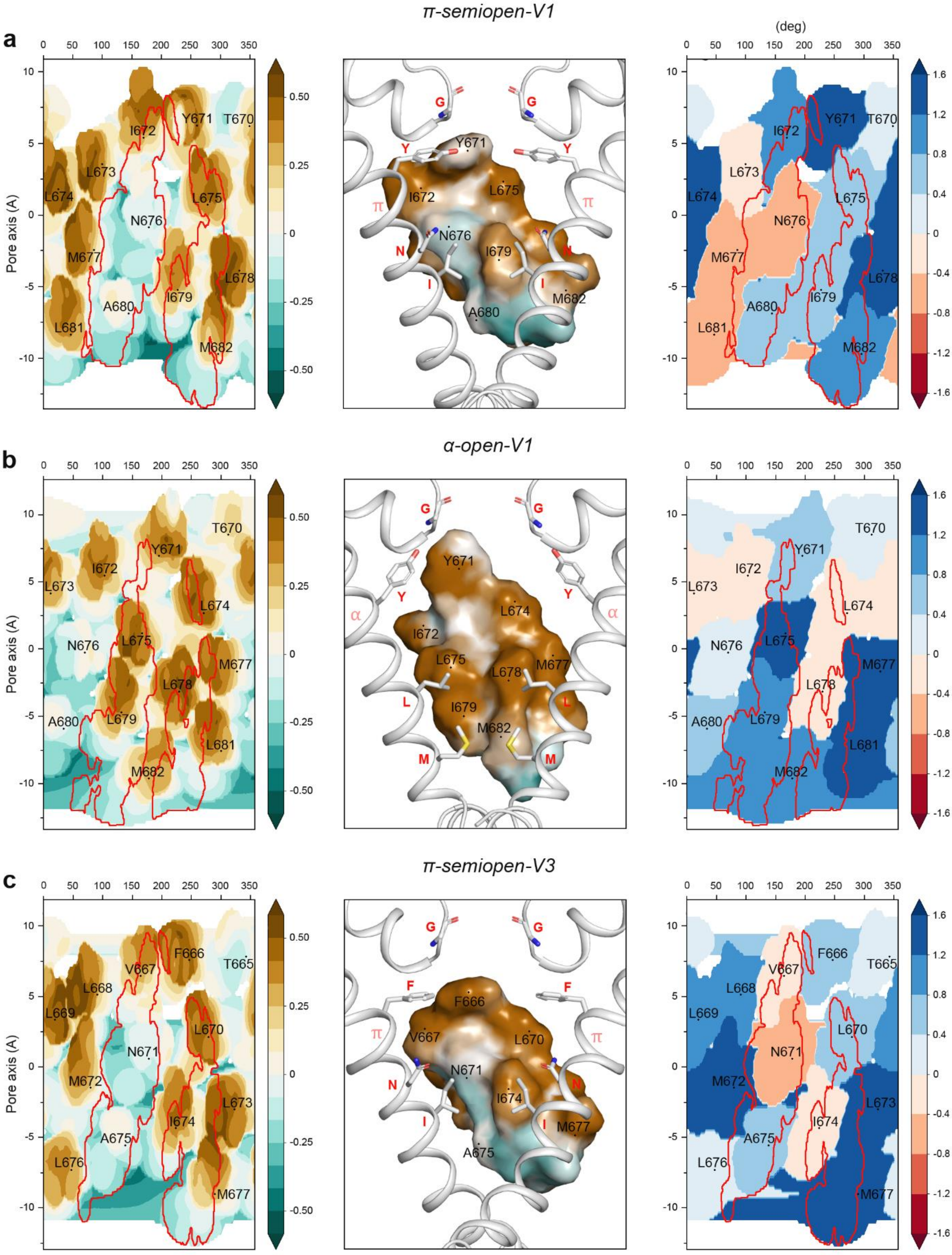

Supplementary Fig. 3 | Contact interfaces between S6 helices at the gate region of rat TRPV1 and mouse TRPV3. a – π-semiopen-V1, **b** – α-open-V1, **c** – π-semiopen-V3. All designations are the same as in Fig3.

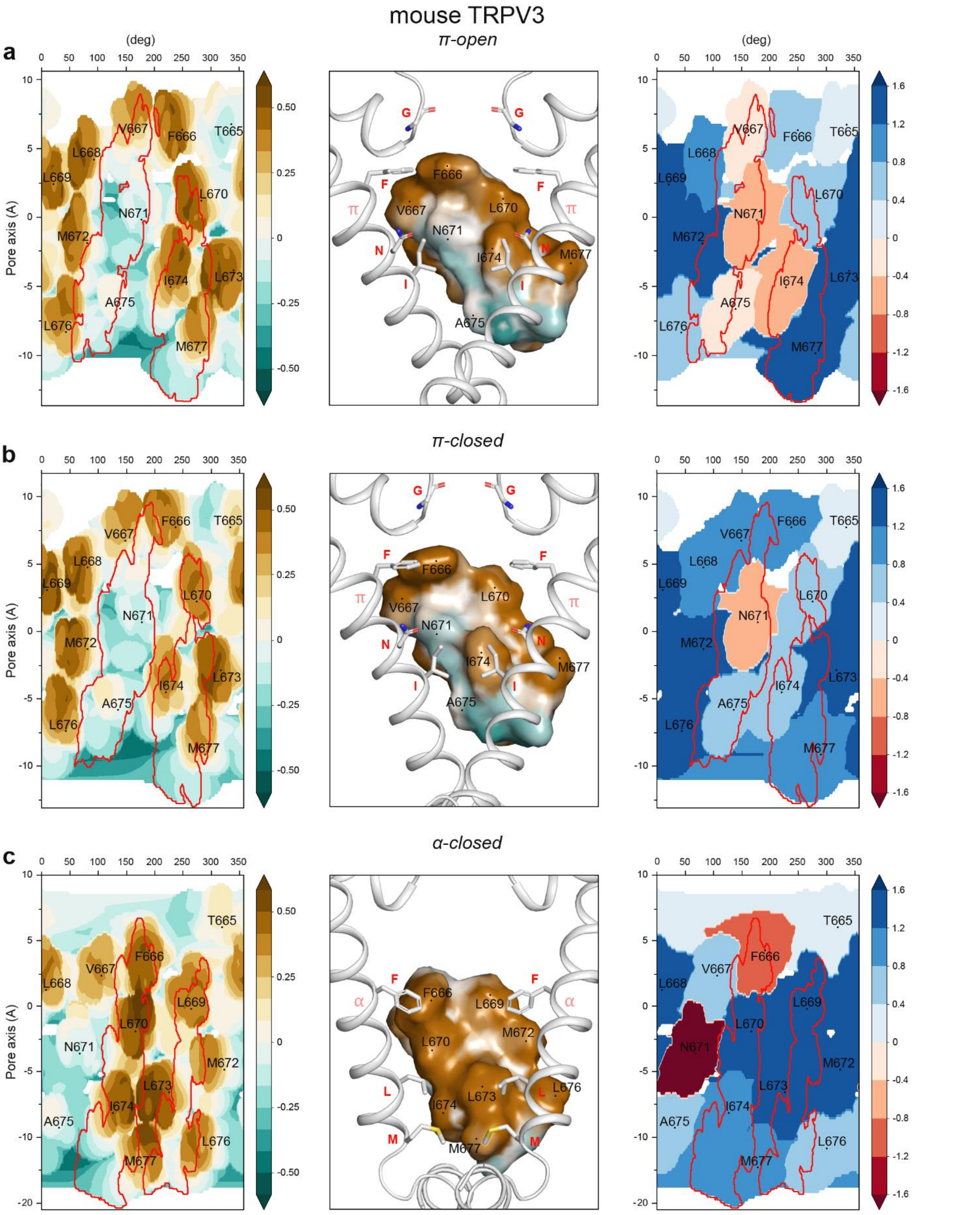

Supplementary Fig. 4 | Contact interfaces between S6 helices at the gate region of mouse TRPV3. **a** – π-open-V3, **b** – π-closed-V3, **c** – α-closed-V3. All designations are the same as in Fig.3.

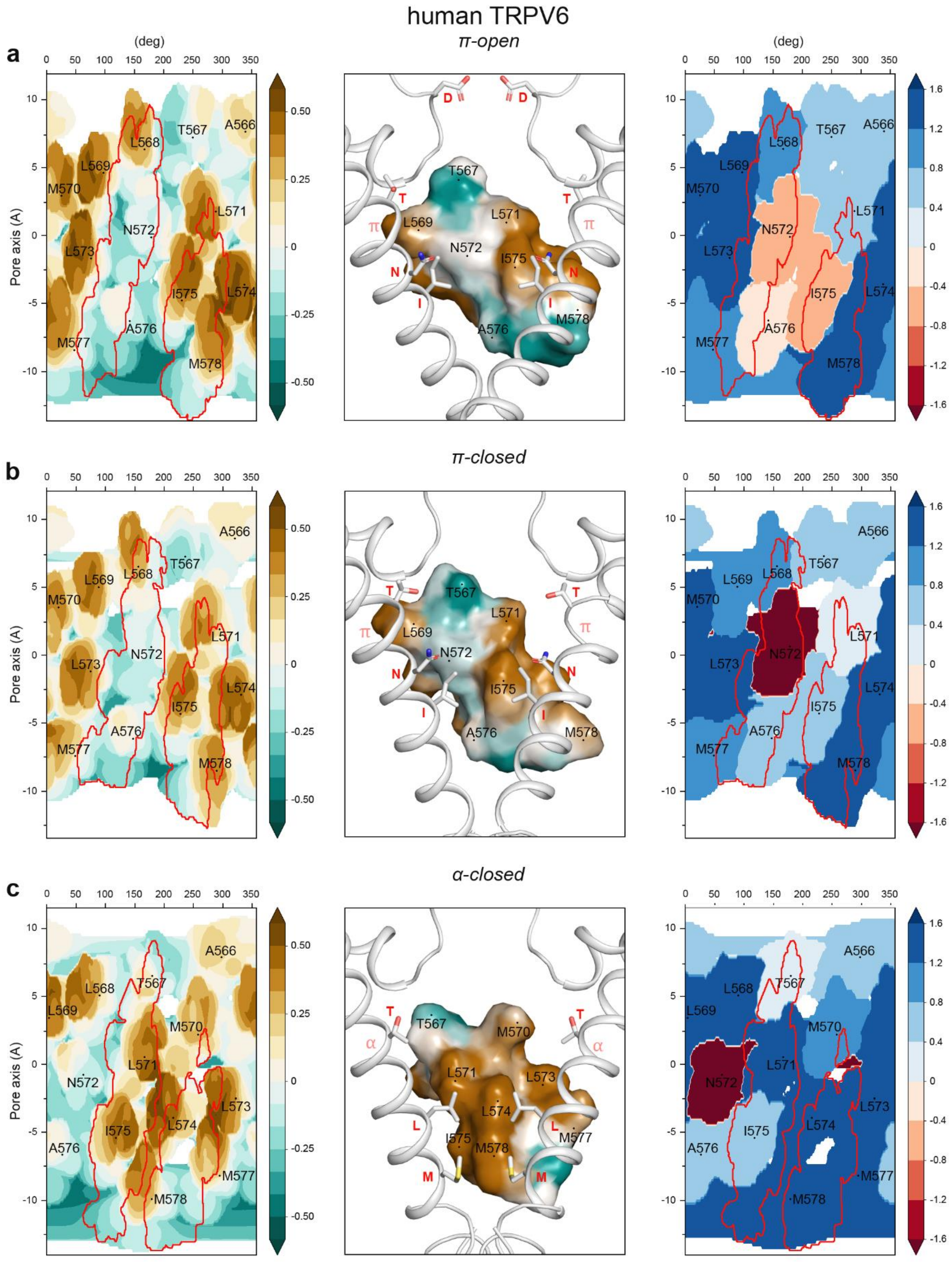

Supplementary Fig. 5 | Contact interfaces between S6 helices at the gate region of human TRPV6. **a** – π-open-V6, **b** – π-closed-V6, **c** – α-closed-V6. All designations are the same as in Fig3.
