# Supplementary material for: Dynamic molecular portraits of ion-conducting pores characterize functional states of TRPV channels
Source: Commun Chem. 2024 Jun 1;7:119. doi: 10.1038/s42004-024-01198-z (PMC11144267; doi:10.1038/s42004-024-01198-z)
Supplement: Supplementary file 2 — Description of Additional Supplementary Files [file 42004_2024_1198_MOESM2_ESM.docx]

Description of Additional Supplementary Files

File name- Supplementary Data 1 –

File description- initial and final configurations in MD simulations

File name- Supplementary Data 2 –

File description- numerical source data for graphs
