# Supplementary material for: Dynamic molecular portraits of ion-conducting pores characterize functional states of TRPV channels
Source: Commun Chem. 2024 Jun 1;7:119. doi: 10.1038/s42004-024-01198-z (PMC11144267; doi:10.1038/s42004-024-01198-z)
Supplement: Supplementary file 3 — Supplementary Data 1 [file 42004_2024_1198_MOESM3_ESM.zip › Supplementary Data 1/List of MD states.docx]

Table 1 List of MD states in Supplementary Data 1,

initial states – MD systems before energy minimization, final states – MD systems after 200 ns production run

| File name | state | system | PDB ID |
| --- | --- | --- | --- |
| TRPV1 | | | |
| pi_openV1_init | initial | π-open-V1 | 7L2W |
| pi_openV1_final | final |  |  |
| pi_closedV1_init | initial | π-closed-V1 | 7L2P |
| pi_closedV1_final | final |  |  |
| pi_semiopenV1_init | initial | π-semiopen-V1 | 7MZC |
| pi_semiopenV1_final | final |  |  |
| alpha_closedV1_init | initial | α-closed-V1 | 7MZD |
| alpha_closedV1_final | final |  |  |
| alpha_openV1_init | initial | α-open-V1 | 7L2U |
| alpha_openV1_final | final |  |  |
| TRPV3 | | | |
| pi_openV3_init | initial | π-open-V3 | 7MIO |
| pi_openV3_final | final |  |  |
| pi_closedV3_init | initial | π-closed-V3 | 7MIN |
| pi_closedV3_final | final |  |  |
| pi_semiopenV3_init | initial | π-semiopen-V3 | 7MIL |
| pi_semiopenV3_final | final |  |  |
| alpha_closedV3_init | initial | α-closed-V3 | 6PVL |
| alpha_closedV3_final | final |  |  |
| TRPV6 | | | |
| pi_openV3_init | initial | π-open-V6 | 7S88 |
| pi_openV3_final | final |  |  |
| pi_closedV3_init | initial | π-closed-V6 | 6E2F |
| pi_closedV3_final | final |  |  |
| alpha_closedV3_init | initial | α-closed-V6 | 7S8C |
| alpha_closedV3_final | final |  |  |
